# Chromosome integrity checkpoints in stem and progenitor cells: transitions upon differentiation, pathogenesis, and aging

**DOI:** 10.1007/s00018-018-2891-z

**Published:** 2018-07-31

**Authors:** Andreas Brown, Hartmut Geiger

**Affiliations:** 1 Institute of Molecular Medicine, Ulm University, Life Science Building N27, James Franck-Ring/Meyerhofstrasse, 89081 Ulm, Germany; 2 Division of Experimental Hematology and Cancer Biology, Cincinnati Children’s Hospital Medical Center, 3333 Burnet Ave, Cincinnati, OH 45229 USA

**Keywords:** Mitotic checkpoints, Spindle assembly checkpoint, Decatenation checkpoint, Chromosomal instability, Stem and progenitor cells, Leukemia, HSCs, HSPCs

## Abstract

Loss of chromosome integrity is a major contributor to cancer. Checkpoints within the cell division cycle that facilitate the accuracy and outcome of chromosome segregation are thus critical pathways for preserving chromosome integrity and preventing chromosomal instability. The spindle assembly checkpoint, the decatenation checkpoint and the post-mitotic tetraploidy checkpoint ensure the appropriate establishment of the spindle apparatus, block mitotic entry upon entanglement of chromosomes or prevent further progression of post-mitotic cells that display massive spindle defects. Most of our knowledge on these mechanisms originates from studies conducted in yeast, cancer cell lines and differentiated cells. Considering that in many instances cancer derives from transformed stem and progenitor cells, our knowledge on these checkpoints in these cells just started to emerge. With this review, we provide a general overview of the current knowledge of these checkpoints in embryonic as well as in adult stem and progenitor cells with a focus on the hematopoietic system and outline common mis-regulations of their function associated with cancer and leukemia. Most cancers are aging-associated diseases. We will thus also discuss changes in the function and outcome of these checkpoints upon aging of stem and progenitor cells.

## Principles of mitotic checkpoint signaling

One major feature of hematopoietic neoplasms is chromosomal instability (CIN). In 95% of all cases bone marrow (BM) cells of chronic myeloid leukemia (CML) patients, for instance, are present with the Philadelphia chromosome arising from a translocation between chromosomes 9 and 22 [1]. Moreover, cells from most acute myeloid leukemia (AML) subsets contain cytogenetic aberrations, such as *t*(8;21) or *t*(15;17) [2]. These findings emphasize the importance of cellular mechanisms that limit CIN to restrict disease initiation. Therefore, a great variety of crucial mechanisms supporting the process of sister chromatid separation and chromosome stability is active before, during, or after mitotic progression of the cell division cycle, such as the spindle assembly checkpoint (SAC) and the decatenation checkpoint (DC). Whereas the SAC is only active in (pro)metaphase and disabled shortly before anaphase onset in the course of mitotic or meiotic progression, the DC is triggered during the late phase of G2 of the cell cycle. Together, both mechanisms prevent chromosomal anomalies, such as translocations, trisomies, and insertions/deletions, hence ensuring genomic integrity of daughter cells upon cell division. In addition, other pathways such as the G1 tetraploidy checkpoint, sometimes referred to as post-mitotic checkpoint, or signal cascades that facilitate precise centrosome duplication have been described: these mechanisms have been considered to contribute to chromosomal integrity as well, although their exact implications in mammalian cells have been discussed [3, 4].

**Fig. 1:**
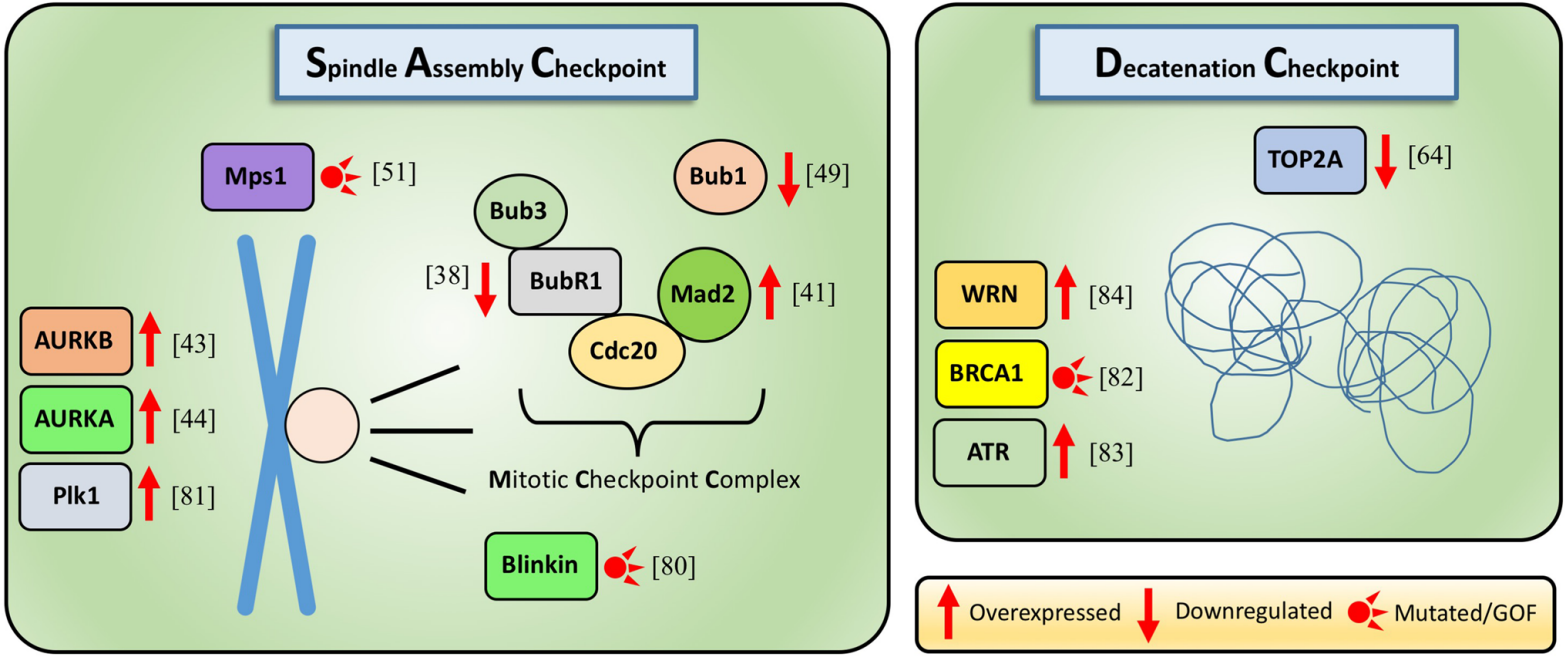
Genes involved in the spindle assembly checkpoint or decatenation checkpoint activity and their deregulation in hematological malignancies

Here, we focus on the current knowledge of the mechanisms of these late G2, mitotic and post-mitotic checkpoints in stem and progenitor cells and highlight differences in comparison with cell lines and differentiated cells. We also present the current understanding of how these checkpoints are altered in diseases and upon aging. We omit other checkpoint mechanisms of genome integrity, such as DNA damage response pathways, which are already discussed elsewhere in detail [5–7]. Figure 1 lists various known mitotic checkpoint alterations and their involvement in hematopoietic malignancies and other types of cancers. They will be discussed in the subsequent paragraphs.

## The SAC ensures fidelity of chromosome segregation in stem and progenitor cells

The SAC is critical for temporarily arresting mitotic progression to enable the accurate coordination between kinetochores and the spindle apparatus or to resolve erroneous chromosomal attachments: sister chromatids must not be separated until all kinetochores are completely and tightly aligned at the metaphase plate. Only in this scenario, the SAC is switched off to finally trigger sister chromatid segregation. If the spindle defects are too severe, though, apoptosis is initiated by p53-dependent and -independent pathways [8, 9]. The SAC involves the inhibitory activity of the mitotic checkpoint complex (MCC) which includes, among others, Cell division cycle 20 (Cdc20), one of the main co-activators of the anaphase promoting complex (APC/C). In response to the recognition of imprecise or false microtubule–kinetochore attachments, several kinetochore components are phosphorylated by Monopolar spindle protein 1 (Mps1) and Aurora B, among others, and MCC component Mitotic arrest deficient 2 (Mad2) rapidly adjusts its native conformation [10, 11]. This switch provokes a signaling cascade that is distributed throughout the nucleus, forcing Cdc20 to incorporate within the MCC. Upon satisfaction of the SAC, Cdc20 is released—now able to stimulate the APC/C [12]. This multi-subunit E3-ubiquitin ligase targets securin (Pttg1) and cyclinB1, the two mayor inhibitors of separase for their proteasomal degradation [13]: being unaffected by its negative regulators, separase catalyzes the site-specific proteolysis of the centromeric cohesion component Scc1 (Rad21): anaphase onset is initiated. This whole process requires tight regulation. If severe DNA aberrations or unequal chromosome numbers remain undiscovered after metaphase, they will be distributed to daughter cells and ultimately contribute to cancerogenesis [14]. The activity of the SAC and the expression of associated genes have been demonstrated in many tissues of the human body—from fast growing mammary epithelial cells to germ cells undergoing meiotic division [15, 16]. Thus, the molecular mechanisms of the SAC have been extensively studied, however, mainly in yeast or cancer cell lines, such as HCT116 and Hela. Here, often unnatural karyotypes, aneuploidies or large chromosome numbers are present, making a functional SAC inevitable, even for these highly transformed cells [17]. However, information on the SAC in untransformed somatic tissues and particularly in (adult) stem cells has lagging behind, with novel and exciting data though emerging over the last couple of years.

In one of the first studies regarding this topic, Rohrabaugh and co-workers demonstrated that hematopoietic stem and progenitor cells (HSPCs) arrest at the G2/M boundary in response to treatment with the spindle drug nocodazole which blocks tubulin polymerization and induce cell death [18]. Interestingly, in murine embryonic stem (ES) cells SAC activity was demonstrated, though apoptosis was not induced in response to prolonged exposure to nocodazole which activated the checkpoint. This behavior might allow for a high tolerance to chromosomal abnormalities and polyploidy, according to the authors, and may at least in part account for the appearance of trisomic disorders [19]. We recently confirmed the presence of the SAC in HSPCs [20] and demonstrated that it is at least in part required for proper engraftment of progenitors in peripheral blood and hematopoietic colony formation. Similar to differentiated cells but in contrast to murine ES cells, prolonged treatment with anti-mitotic drugs, such as nocodazole or taxol to activate the checkpoint causes p53-dependent apoptosis. Analogous to other types of cells, the SAC depends on the activity of Mps1 and Aurora B since chemical inhibition of these components caused a checkpoint override as assessed by cell cycle analysis. Strikingly, in hematopoietic stem cells (HSCs), inhibition of the SAC, while present, had only mild effects, as chemical inhibition with the Mps1 inhibitor reversine neither increased the number of chromosomal aberrations nor did it negatively influence late engraftment after competitive transplantation (a feature, which has been shown to be mainly driven by HSCs [21]). In contrast, the SAC was shown to be of critical importance for more committed hematopoietic progenitors, such as Lin-cKit+ cells. Here, checkpoint inhibition induced a massive failure to form colonies on methylcellulose and negatively influenced their engraftment potential [20]. Interestingly, in murine HSPCs the SAC only in part depends on the MCC component Bub1-related kinase (BubR1), as *BubR1*-haploinsufficiency did not alter cell cycle dynamics and the clonal colony formation ability of HSPCs [20]. Another study revealed that HSCs hypomorphic for *BubR1* (Bub1b^H/H^) lose their engraftment potential upon secondary transplantation in recipient mice and after tertiary transplantation Bub1b^H/H^ donor-derived HSCs completely failed to engraft. These findings unveil that, ultimately, *BubR1* deficiency indeed causes premature HSC exhaustion, but only after several rounds of cell division, consistent with the reported minor role of that gene for the SAC in HSCs [22]. Interestingly, upon overexpression of BubR1 in mice, aneuploidy and the incidence of age-related cancers were decreased, whereas overall lifespan was increased [23, 24]. Consequently, BubR1 may also account as a lifespan gene [25]. Another study addressed SAC function in human hematopoietic progenitors: here, the authors conducted experiments in BM cells from *Mad2* haploinsufficient (*Mad2*^+*/*−^) mice and examined absolute numbers as well as cell cycle dynamics of various hematopoietic subpopulations. Although mature progenitors, such as granulocyte precursors exhibited a normal behavior in terms of cycling activity and absolute cell numbers, large alterations in absolute cell numbers, high apoptosis rates and enhanced proliferation upon cytokine stimulation of immature progenitors were observed. Interestingly, the authors revealed that in human hematopoietic progenitors SAC component Mad2 associates with c-Kit, an important receptor tyrosine kinase of HSPCs [26]. Similar observations with respect to Mad2 were reported for skin cells: upon depletion of *Mad2*, the hair follicle bulge stem cell pool was diminished, while there were no large differences in term of function and numbers of interfollicular epidermal cells. However, these mice lost most of their hair after birth, and furthermore, chromosomal analysis of the epidermis revealed high amounts of aneuploidy, a known consequence of SAC deficiency [27]. The study further supported the concept that an impaired SAC has different outcomes on various cells of the same tissue. Last, the activity of Mad2 has been proposed to also affect asymmetric/symmetric cell division of stem cells by influencing their spindle positions during mitosis. Hence, SAC component Mad2 might be a novel factor involved in controlling stem cell differentiation and self-renewal [26, 28]. A major result of abrogated SAC activity is the occurrence of trisomic disorders. Indeed, it is assumed that trisomic cells present with an inherent defect in the SAC. Pfau et al. described that fetal HSCs isolated from mice carrying constitutional trisomies of chromosome 11 or 16 displayed severe engraftment defects [22]. The activity of the SAC could also be demonstrated in muscle progenitor and stem cells (satellite cells): upon tamoxifen-mediated knockdown of Mps1, these cells failed to differentiate and were unable to expand [29]. In murine megakaryocytes, which are polyploid and have chromosomes numbers of up to 256N, the SAC has a crucial impact on megakaryopoiesis in terms of number of splenic megakaryocytes while checkpoint failure triggered by *BubR1* knockout did not result in a significant lack of thrombocytes [30].

The SAC has been demonstrated to be present in stem cells from other distinct organisms as well: in *Drosophila* embryos downregulation of the SAC by deletion of *Mad2* caused massive mitotic failure and depletion of the neuronal progenitor cell pool as well as medulla reduction and enlarged central brain sizes [31]. In malignant fly tumor neural stem cells, deregulation of the SAC by genetic knockdown provoked impairment of sister chromatid segregation and aneuploidy. Crucially, these stem cells were not able to form colonies anymore. Disruption of SAC component Aurora A, however, did not lead to inhibition of aneuploidy although the SAC was inhibited [32, 33]. Last, in *C. elegans*, SAC inhibition by Mad2 knockdown in germline stem and progenitor cells triggers a corrupted spindle assembly in terms of spindle length and a delay in mitotic progression. Interestingly, whereas *Mad2*, *BubR1* and *Bub3* are reported to be conserved between *C. elegans* and higher organisms, a homolog for the checkpoint kinase Mps1 was not found in this nematode [34]. Together, these findings support the conclusion that the SAC is of general importance for stem and progenitor cells in most organisms. In higher organisms, especially in mammals, the pool of data suggests that the lack of correct activation of the SAC has varying outcomes, depending on the tissue and the affected SAC-related genes.

## Changes in SAC signaling in cancer

Chromosomal instability provokes both tumor initiation and progression, especially in hematopoietic neoplasms. Usually, the underlying cells driving leukemias such as acute myelogenous leukemia (AML) or CML are malignantly transformed HSPCs [35–37]. Strikingly, many studies could demonstrate the abrogation or malfunction of the SAC in leukemia cells taken from patients. For instance, it has been shown that in most BM samples from AML patients, the important SAC regulator *BubR1* is downregulated, whereas other SAC-involved genes such as budding uninhibited by benzimidazoles 1 (*Bub1*) and *Bub3* were not mis-regulated [38]. The reduced expression of *BubR1* goes along with a strong deregulation of the SAC in these cells: The two main regulators of separase, cyclinB1 and securin are prematurely degraded and high levels of chromosomal aberrations, such as trisomies were observed. However, in response to overexpression of *BubR1,* SAC activity and sensitivity to nocodazole can be regained. Previously SAC-deficient cells stabilize their cyclin B1 amounts after treatment with anti-mitotic drugs and the frequency of chromosome mis-segregation is decreased, whereas apoptosis levels are elevated [38]. Another SAC component, Mad2, which is required for instantly distributing the SAC signal throughout the nucleus, has been described to be mis-regulated in leukemia as well [39, 40]. Indeed, overexpression of *Mad2* in transgenic mice promoted aneuploidy, anaphase bridges, chromosome breaks as well as initiation of tumors, such as lymphoma and hepatocellular carcinoma. Interestingly, elevated levels of Mad2 did not appear to have an impact on further tumor progression, suggesting that Mad2 mis-regulation is primarily involved in initial steps of tumor formation [41]. Moreover, in leukemic (AML) cells positive for the fusion gene AML-ETO (AEtr), it was demonstrated that the SAC was deregulated since these cells failed to arrest in response to anti-mitotic drugs. In such cells, *BubR1* levels were reduced upon nocodazole treatment, causing mis-regulated APC/C activity and hence premature securin degradation. Interestingly, other checkpoint proteins, such as Mad2 or Bub3 were not downregulated, indicating a specific correlation of the presence of AEtr and decreased BubR1 expression [42]. Further on, expression levels of the SAC components Aurora A/B have been reported for AML cells [43], whereas upregulation of Aurora A has also been connected to the initiation of myelodysplastic syndrome (MDS) [44]. This heterogeneous class of various blood cancers also in most cases emerges from transformed HSPCs [45, 46]. The van Deursen laboratory showed that overexpression of the MCC regulator *Bub1* in mice induced tumor formation [47], while the level of expression of the gene was reduced in AML specimens and cell lines, such as K562 and HL60 [48, 49]. Other analyses revealed high expression of Mad2, Aurora B and Cdc20 in MDS. Interestingly, each MDS subtype was reported to have its own expression profile of SAC genes. High expression, especially of Mad2 and Cdc20 was associated with thrombocytopenia and an overall poor survival rate [45]. That implies that the level of impairment of the SAC triggered by Cdc20 and Mad2 mis-regulation might directly contribute to the severity of the disease. In Runt-related transcription factor 1 (RunxX1)-mutated acute lymphoblastic leukemia (ALL) cells, severe SAC malfunctions were also described. With the SAC regulator Mad2 being downregulated, these cells failed to sufficiently arrest upon anti-mitotic drug treatment and ALL-derived cells displayed high amounts of trisomies and other mayor chromosomal abnormalities [50]. Mutations in the important MCC regulator Mps1 have been implied in the initiation of ALL and AML as well, and indeed, inhibitors targeting Mps1 are already in clinical trials [51, 52]. Finally, the checkpoint has also been studied in transformed induced pluripotent stem (iPS) cells that induced aggressive teratomas [53]: these teratoma cells displayed aberrant cell cycle regulation and CIN, which could be linked to high expression of the checkpoint kinase Aurora A. Indeed, co-inhibition of Aurora A during iPS generation prevented the transformation process [54]. These reports demonstrate that transformed stem cells can also exhibit high levels of expression of genes important for SAC function that contribute to pathology.

In summary, a broad range of hematological malignancies, such as MDS, AML and ALL, have been reported to be present with failed or mis-regulated SAC activity, primarily due to mis-expression (both higher and lower than normal) of genes related to SAC activity. The data imply that chromosomal aberrations such as CIN and cell cycle deregulation in cancer might be a direct consequence of SAC malfunction in stem and progenitor cells, since these cells are very often the driver of these diseases.

## DC and tetraploidy checkpoint mechanisms and their relevance in stem cells and cancer

The decatenation checkpoint (DC) is already active in the late G2 phase, the third period of interphase, and thus earlier than the SAC. In this phase of the cell cycle, sister chromatids, though still being decondensed, must already be physically connected by cohesin ring complexes, whereas the chromatin of complementary strands must not be intertwined. Upon entanglement, further cellular progression is blocked, enabling the physical separation of these DNA strands by the activity of topoisomerase II α (TOP2A) [55–57]. The DC is not directly related to the G2/M checkpoint that is exclusively activated in response to DNA damage; however, both mechanisms share a distinct set of signaling proteins, such as Ataxia telangiectasia and Rad3-related protein (ATR) and Polo-like kinase 1 (Plk1) [58]. It has been claimed that the DC is absent in murine ES and neural progenitor cells as well as human hematopoietic progenitor (CD34 +) cells as stem cell multipotency may not be compatible with proper DC activity. Upon differentiation of ES cells into more committed progenitors, interestingly, the checkpoint regained activity [59]. Inversely, whereas the activity of this checkpoint was demonstrated in a broad range of cell lines and differentiated tissues, it was absent in various carcinoma and lung cancer cell lines, such as A549 and ACC-LC-172 [60–62]. Therefore, it is discussed that the absence of the DC may be a driver of cancer progression [63], or, it might be an indicator of the stem cell-like character of the cells underlying the cancer. Indeed, also AML cells do not activate the DC in response to entanglement of chromosomes. Moreover, AML cells show constitutive high expression of the protein Metnase, which is able to support TOP2A function by its histone methylation activity forcing chromosome decatenation without the cell having to arrest [64]. Not surprisingly, even in the presence of TOP2A inhibitors AML cells are able to promote decatenation without an arrest at G2, while reduction of Metnase levels can re-activate DC activity in AML cell lines [64, 65]. DC deficiency has been also reported for most melanoma cell lines. However, in these cells inhibition of TOP2A cannot be compensated by Metnase, as in AML cells, provoking cell cycle arrest and apoptosis [66]. Similarly, colon cancer cells, such as HCT116 and HT-29, which have a defective G2 decatenation checkpoint as well, can be eliminated by TOP2A inhibitors. [67].

In conclusion, the data published imply that DC activity is especially important for differentiated cells and that DC deficiency might be a general feature of cancer cells, whereas no evidence exists so far with respect to its activity in primitive and undifferentiated cells. Its absence may thus be especially involved in tumor progression. However, additional research is mandatory to address the role of the DC especially in stem and progenitor cells.

The post-mitotic checkpoint, also termed G1 tetraploidy checkpoint, was reported to arrest cells which suffered from severe spindle defects resulting from premature mitotic exit or SAC failure p53-dependently finally in early G1 [68]. This pathway is, therefore, deregulated in cancer cells with reduced or absent p53 activity [55]. Some researchers claim that also mature and untransformed mammalian cells do not activate this checkpoint [3, 4]. Research from our laboratory suggested the presence of this checkpoint at least in HSPCs. Upon inhibition of cycling of HSPCs with the specific Mps1 inhibitor reversine, we observed a strong and durable G1 arrest. Furthermore, reversine-treated progenitor cells were not able to form colonies on methylcellulose anymore, implying a permanent cell cycle arrest. This finding demonstrated the presences of a post-mitotic checkpoint blocking HSPCs with corrupted SAC activity from further cycling [20]. Similarly, the group of Andrew Brack described the existence of a p21^Cip1^(Cdkn1a)-dependent post-mitotic checkpoint in muscle stem and progenitor cells, also activated by Mps1 inhibition [29]. According to the authors, this mechanism may be involved in preventing early malignant transformation of stem and progenitor cells.

Finally, a small number of reports already addressed a particular checkpoint arresting cells at the G1/S boundary in response to loss of centrosome integrity which includes improper centrosome numbers, for instance. Key players of this checkpoint are p53 and p21, analogous to the G1/S DNA damage response [69]. Whether this mechanism is active in stem and progenitor cells and whether it has an impact during cancer initiation or progression has not yet been studied, though.

## Changes of mitotic checkpoint signaling during the aging process

Cancer is an aging-associated disease [70]. In general, our knowledge on aging-associated changes in the function of mitotic checkpoints is very limited. One paradigm claims that an increase in the number of DNA mutations upon aging is linked to the higher incidence of cancer in the elderly [71]. However, while the number of single point mutations found in BM samples from AML patients increases linearly with age, the number of AML incidents raises exponentially during the same time frame [72]. This discrepancy implies that deregulation of regulatory pathways, such the SAC, might in theory also play a role in cancer initiation. One report, however, suggested that there is no correlation between SAC activity and longevity and that the reliability and function of the SAC decline rather with the number of mitotic divisions and not primarily with age [73]. Human cells probably maintain a very vigorous SAC upon aging since a direct correlation between checkpoint robustness and body mass was described [74]. Further findings suggest that aneuploidy in aged mouse eggs is not primarily caused by a defective SAC, in contrast to studies from other research groups [18, 75]. Moreover, the group of Andrew Brack reported that although the SAC is essential for maintaining muscle stem cell function, aging does not have an impact on the robustness of the SAC itself [29]. Inversely, there is a correlation between age and expression of the checkpoint genes Mad2, Aurora B and Cdc20 in myelodysplastic syndrome (MDS), an characteristic aging-associated dysplasia of the blood-forming system. The same study also demonstrated a correlation of expression levels of these genes and the developmental stage of MDS, as already discussed in the previous paragraph [45].

Mechanistically, expression of *BubR1* has been reported to continuously decline upon aging in several tissues such as ovary and testis, whereas its expression in other tissues or transcription of different checkpoint genes such as *Bub3* were reported to be unaltered [76]. High expression of the SAC component BubR1 has been linked to longevity as well as to low rates of chromosomal aberrations and aneuploidy. Upon overexpression of *BubR1* in vivo, direct consequences of low SAC activity, such as false microtubule–kinetochore attachments, could be avoided [25].

**Fig. 2:**
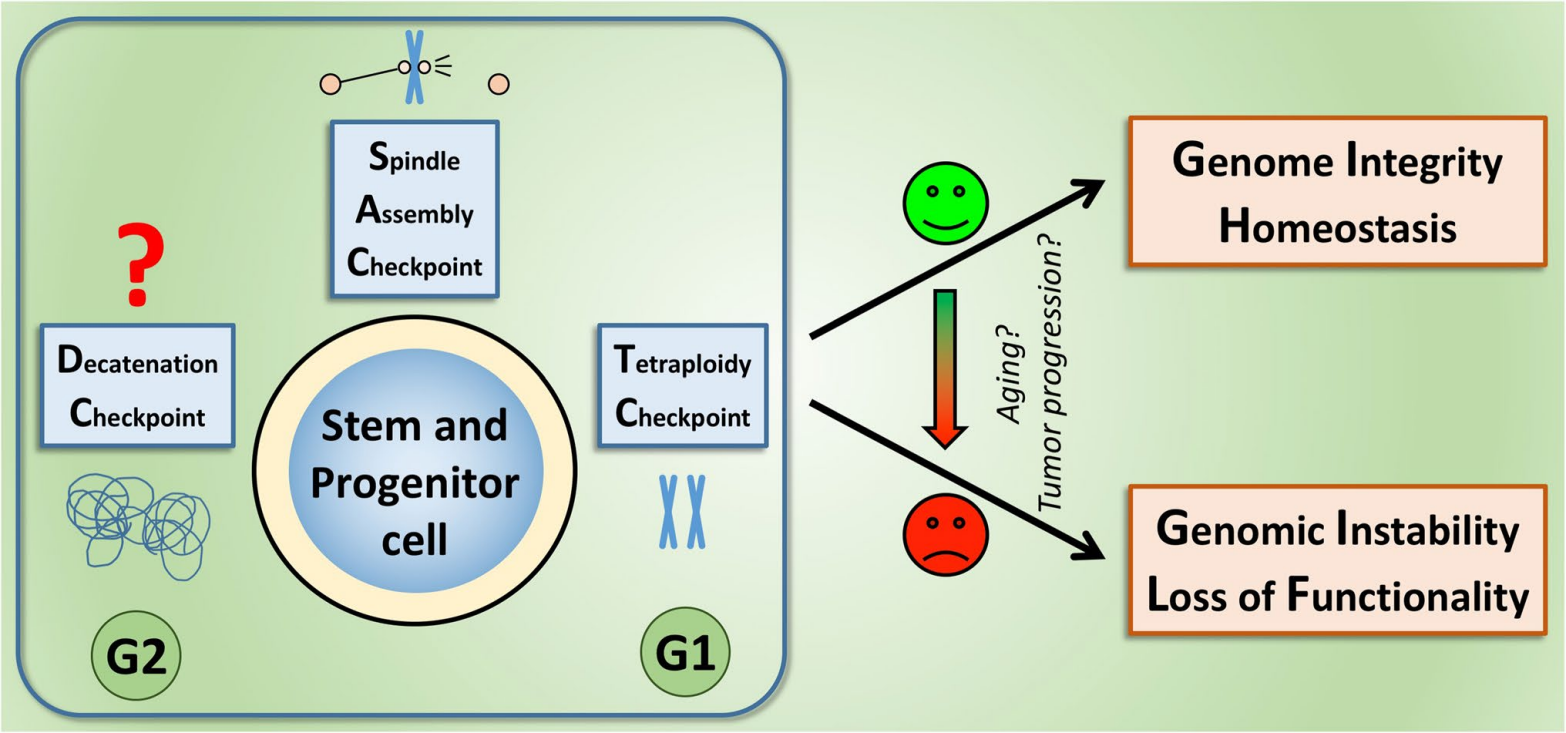
Mechanisms that ensure the fidelity of chromosome segregation, especially the SAC are required to maintain genome integrity and homeostasis of stem and progenitor cells. Whereas a growing number of studies demonstrate the activity of the SAC and the tetraploidy (post-mitotic) checkpoint in these cells, it is not clear yet, whether they also activate a pathway homologous to the DC. Strikingly, many reports illustrate that without these mechanisms, stem and progenitor cells may take the route towards chromosomal instability, provoking loss of functionality, transformation and, ultimately, cancer

In summary, the relative robustness of the SAC indicates that checkpoint deregulation may not be an early event during carcinogenesis but might rather be involved in tumor progression (as suggested in Fig. 2). Whether there is a role in the elevated initiation of cancer in the elderly needs to be further investigated, as the published data do not allow for unequivocal conclusions on: (a) the extent to which aging affects mitotic checkpoints and (b) whether SAC alterations contribute to the increase of cancer in the elderly. Other checkpoints, such as DNA damage response pathways, indeed have been reported to change upon aging [77], and might thus be also involved in the contribution to aging-associated cancers.

## Directions

Our knowledge on mitotic checkpoint signaling in stem and progenitor cells is still limited but over the recent years novel findings have started to shed light on the mechanisms that govern it and its importance for health and disease. Studies that investigate these checkpoints in HSPCs, muscle satellite cells, neuronal and ES cells have been providing initial insights in their function. This is especially relevant since many reports demonstrate the abrogation of the SAC or the DC, especially in hematopoietic neoplasms, such as MDS, CML and AML but also other types of cancers, such as lung cancer. In these cases, cancer most likely emerges from transformed stem and progenitor cells. Here, very often chromosomal disorders, such as translocations or aneuploidies, are found—one major consequence when mitotic checkpoints fail to operate reliably. These results emphasize the importance of investigations of mitotic checkpoint signaling in still untransformed stem and progenitor cells and the contribution of failing checkpoints to cancer initiation and progression, which might allow to therapeutically target such checkpoints. Indeed, it was demonstrated that re-activation of the SAC in AML cells promotes sensitivity to chemotherapeutics such as taxol [38]. In other cases, inhibition of Aurora kinases which were found to be overexpressed in cancer cells promoted apoptosis [78, 79]. Finally, the re-activation of the DC in AML-derived cell lines was able to induce cell cycle arrest and apoptosis. Consequently, further research into the function of these checkpoints in both young and aged stem and progenitor cells may allow the development of novel pharmaceutical concepts for the treatment of cancer.
